# Antibiotic prescribing in neonatal intensive care units of two tertiary care hospitals at central India

**DOI:** 10.1186/2047-2994-4-S1-P174

**Published:** 2015-06-16

**Authors:** M Sharma, C Hauge, J Mandaliya, G Marrone, C StålsbyLundborg

**Affiliations:** 1 Public Health Sciences, Global Health (IHCAR), KarolinskaInstitutet, Stockholm, Sweden; 2 Pharmacology, R D Gardi Medical College, Ujjain, Madhya Pradesh, India; 3 Pediatrics, R D Gardi Medical College, Ujjain, Madhya Pradesh, India

## Introduction

Neonates are vulnerable for systemic infections and wise use of antibiotics is recommended for the group to cut down the risk of development of antibiotic resistance, a global threat. There is a general lack of studies that present antibiotic prescribing practices at neonatal intensive care units (NICUs) specifically from the countries with high birth rate such as India.

## Objectives

To compare and describe the situation of antibiotic prescribing at neonatal intensive care units of two private, tertiary care hospitals of Ujjain district in Central India.

## Methods

A cross-sectional study was conducted from 2008 till 2011using customized form. Antibiotics were classified based on WHO anatomical therapeutic chemical (ATC) classification and diagnoses were classified using International Classification of Diseases-10.

## Results

Of total 1789 neonates, 1572 were admitted at the NTH and 217 at the TH. Sepsis was most common diagnosis in both hospitals (>30%). Antibiotics were prescribed to higher percentage of neonates with sepsis (NTH-97% and TH-94%) than to the rest (NTH-89%, TH-71%). Most frequently prescribed antibiotics for this group were fixed dose combinations of antibiotics (FDCs, not present in WHO ATC list, 30%) and 3^rd^ generation cephalosporins (28%) in the NTH and 3^rd^ generation cephalosporins and aminoglycosides (36% each) and FDCs (12%) in the TH. The adherence to the WHO’s model list of essential medicines and the Indian national list for children were higher in the teaching hospital than in the non-teaching hospital, 85% and 50% respectively (p<0.01).

## Conclusion

Broad-spectrum antibiotics; including new FDCs were prescribed in both hospitals but extensively at the NTH. The adherence to available essential medicines lists was significantly lower at the NTH. An unnecessary exposure of neonates with the higher classes of antibiotics might be seen as a threat for the development of antibiotic resistance.

There is an urgent need to develop; a diagnoses specific antibiotic prescribing guidelines for neonates and measures to reduce the prevalences of infectious diseases using antibiotic stewardship programs.

## Disclosure of interest

None declared.

